# Comparative metabolic and transcriptional analysis of a doubled diploid and its diploid citrus rootstock (*C. junos* cv. Ziyang xiangcheng) suggests its potential value for stress resistance improvement

**DOI:** 10.1186/s12870-015-0450-4

**Published:** 2015-03-18

**Authors:** Feng-Quan Tan, Hong Tu, Wu-Jun Liang, Jian-Mei Long, Xiao-Meng Wu, Hong-Yan Zhang, Wen-Wu Guo

**Affiliations:** Key Laboratory of Horticultural Plant Biology (Ministry of Education), Key Laboratory of Horticultural Crop Biology and Genetic Improvement (Central Region) (Ministry of Agriculture), College of Horticulture and Forestry Sciences, Huazhong Agricultural University, Wuhan, 430070 China

**Keywords:** Citrus, Doubled diploid, Stress tolerance, Primary and secondary metabolism, Transcriptome

## Abstract

**Background:**

Polyploidy has often been considered to confer plants a better adaptation to environmental stresses. Tetraploid citrus rootstocks are expected to have stronger stress tolerance than diploid. Plenty of doubled diploid citrus plants were exploited from diploid species for citrus rootstock improvement. However, limited metabolic and molecular information related to tetraploidization is currently available at a systemic biological level. This study aimed to evaluate the occurrence and extent of metabolic and transcriptional changes induced by tetraploidization in Ziyang xiangcheng (*Citrus junos* Sieb. ex Tanaka), which is a special citrus germplasm native to China and widely used as an iron deficiency tolerant citrus rootstock.

**Results:**

Doubled diploid Ziyang xiangcheng has typical morphological and anatomical features such as shorter plant height, larger and thicker leaves, bigger stomata and lower stomatal density, compared to its diploid parent. GC-MS (Gas chromatography coupled to mass spectrometry) analysis revealed that tetraploidization has an activation effect on the accumulation of primary metabolites in leaves; many stress-related metabolites such as sucrose, proline and γ-aminobutyric acid (GABA) was remarkably up-regulated in doubled diploid. However, LC-QTOF-MS (Liquid chromatography quadrupole time-of-flight mass spectrometry) analysis demonstrated that tetraploidization has an inhibition effect on the accumulation of secondary metabolites in leaves; all the 33 flavones were down-regulated while all the 6 flavanones were up-regulated in 4x. By RNA-seq analysis, only 212 genes (0.8% of detected genes) are found significantly differentially expressed between 2x and 4x leaves. Notably, those genes were highly related to stress-response functions, including responses to salt stress, water and abscisic acid. Interestingly, the transcriptional divergence could not explain the metabolic changes, probably due to post-transcriptional regulation.

**Conclusion:**

Taken together, tetraploidization induced considerable changes in leaf primary and secondary metabolite accumulation in Ziyang xiangcheng. However, the effect of tetraploidization on transcriptome is limited. Compared to diploid, higher expression level of stress related genes and higher content of stress related metabolites in doubled diploid could be beneficial for its stress tolerance.

**Electronic supplementary material:**

The online version of this article (doi:10.1186/s12870-015-0450-4) contains supplementary material, which is available to authorized users.

## Background

Polyploidy is a common biological phenomenon and plays an important role in evolutionary history of plants [1-3]. Almost all angiosperms have undergone at least one round of whole-genome duplication in the course of their evolution [4,5]. Polyploids are classified into autopolyploids and allopolyploids. The first comes from doubling a diploid genome. And the latter arises from the combination of two or more sets of divergent genomes [6,7]. Many major crop plants including wheat (allohexaploid), cotton (allotetraploid), oilseed rape (allotetraploid), sweet potato (autotetraplooid), rice and maize (paleopolyploid) are polyploids. Moreover, polyploidy cultivars are prevalent in fruit plants, such as banana (triploid), grape (tetraploid), kiwifruit and persimmon (hexaploid), strawberry (octaploid). Phenotypic variations caused by polyploidization possess the potential to improve agricultural productivity and efficiency, especially in increasing biomass and stress tolerance.

Polyploidy has a significant influence on morphology and physiology of newly formed offspring. Compared with the corresponding diploids, autopolyploids tend to have larger cells, which result in the enlargement of single organs, such as leaves, flowers and seeds [8,9]. Physiological traits such as plant height, growth rate, flowering time, and fertility also can be altered by polyploidization [10-12]. It has been shown that tetraploidization might significantly increase stress tolerance [13,14].

A limited number of studies have investigated metabolic changes caused by autopolyploidization, and those studies focused on only specific metabolites [12]. The production of alkaloids was enhanced in artificial autotetraploids *Hyoscyamus niger* [15]. More artemisinin was produced in hairy roots of autotetraploid *Artemisia annua* [16]. Similarly, essential oils were accumulated much more in autotetraploid aromatic grasses (*Cymbopogon*) [17]. Moreover, the concentration of some metabolites like GAs (glycoalkaloids) were differentially influenced by autotetraploidy, increasing the content of minor GAs and decreasing the content of major GAs in autotetraploid *Solanum commersonii* [18].

Gene expression variations caused by allopolyploidization have been widely reported in many species including *Arabidopsis* [19,20], citrus [21], maize [22], and tobacco [23]. However, the studies on autopolyploidization aimed at identifying the alterations of genome expression patterns are relatively less than those on allopolyploidization. It is probably because autopolyploidy has long been viewed as less frequent and less important. The number of the genes differentially expressed between diploid and autotetraploid potato was about 10% [24]. A much lower rate (less than 2%) was observed when autotetraploid *Arabidopsis* was compared with diploid progenitor [25]. Similarly, study performed in autotetraploid and diploid Rangpur lime (*Citrus limonia*) showed about 1% variation in transcriptome [26]. Notably, the differentially expressed genes induced by autotetraploidization were highly related to stress response [14,25].

Citrus is one most important fruit crop in the world. However, citrus production is influenced by many environmental stresses including drought, salinity and extreme temperature [27]. Citrus rootstock improvement is required to cope with these abiotic stresses. Ziyang xiangcheng is a local citrus rootstock originated from southwest China. It was considered a putative hybrid of *Citrus ichangensis* and *Citrus reticulata* [28]. Because of its excellent performance in biotic and abiotic stresses, it has been widely used as a citrus rootstock in China [28,29]. Citrus rootstocks are propagated through polyembryonic seeds and genetically identical to the maternal plant [30-32]. The majority of citrus genotypes are apomictic, and all the apomictic embryos originate from nucellar cells [30]. Tetraploidization events are frequent in apomictic citrus genotypes [30,33]. Doubled diploid seedlings in apomictic genotypes are considered to arise from somatic chromosome doubling of maternal cells and should be genetically identical to the seed source tree [30,31]. Recent studies demonstrate that genome doubling is often considered to confer plants a better adaptability to various environmental stresses [13,14,33,34]. Therefore, doubled diploid citrus rootstocks were expected to have substantial advantage over diploid in stress tolerance. In our previous citrus breeding program, we obtained plenty of spontaneous doubled diploids from various citrus rootstock varieties, including Ziyang xiangcheng (*Citrus junos* Sieb. ex Tanaka) [35,36].

To test the effects of tetraploidization on Ziyang xiangcheng, we performed comparative metabolic and transcriptional analysis of doubled diploid and its diploid parent. Our results revealed that doubled diploid Ziyang xiangcheng had a distinct metabolic phenotype, compared with diploid. Many stress related metabolites such as sucrose, proline and GABA were enhanced in doubled diploid. However, less than 1% of genes were differentially expressed between doubled diploid and its diploid parent. Interestingly, these differentially expressed genes were highly related to stress response.

## Results

### Ploidy determination and analysis of genetic constitution

Eight uniform 4× seedlings out of previously identified fifteen doubled diploids were selected and further verified by flow cytometry. These eight 4× seedlings together with thirteen 2× seedlings were then analyzed by the SSR markers. All the SSR makers revealed that the eight 4× and nine 2× plants possessed the same alleles (Additional file 1). This signified that the 4× seedlings derived from genome doubling of the 2× genotype. And three diploids with heterozygous loci (Additional file 1) were excluded for further study.

### Morphological changes following tetraploidization

**Figure 1:**
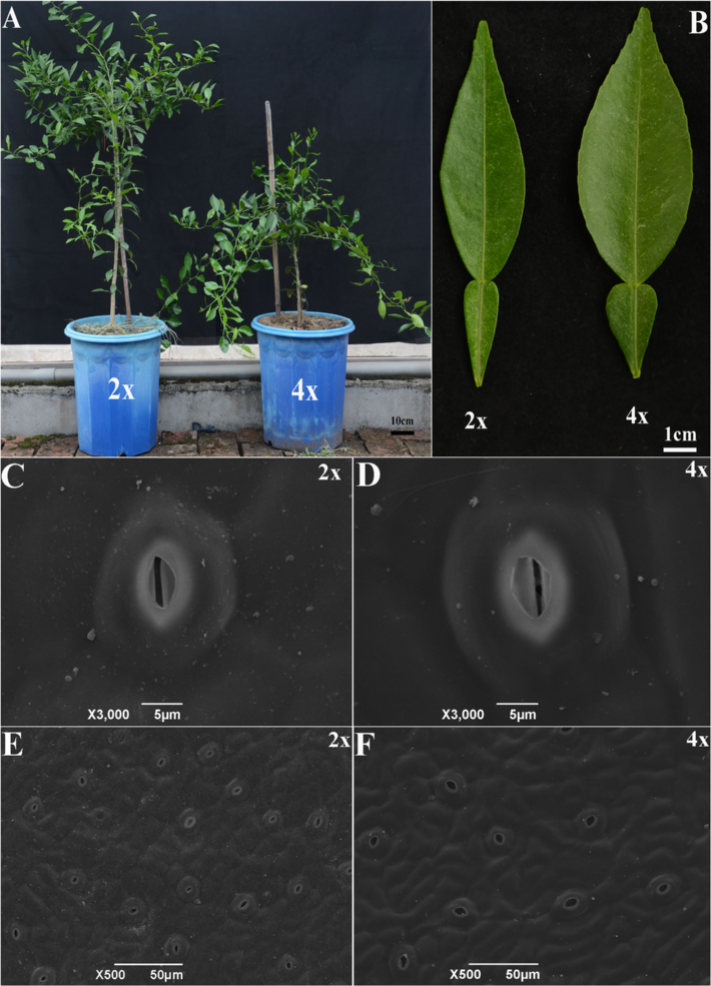
**Morphological characterization of 2× and 4× Ziyang xiangcheng. (A)** 2× and 4× seedlings; **(B)** Leaves of 2× and 4×; **(C)**, **(D)** Stomata size of 2× and 4×; **(E)**, **(F)** Stomata density of 2× and 4×.

In order to investigate morphological changes caused by tetraploidization, morphological analysis on plant height, stem diameter, leaf area, leaf thickness, stomata size and density was conducted. Compared to 2×, 4× has typical tetraploid morphological features, such as shorter plant height, larger and thicker leaf, larger stomata size and lower stomata density (Figure 1 and Additional file 2). Additionally, enlargement in leaf structure of 4x was observed by anatomical analysis (Additional files 3 and 4).

### Changes of primary metabolic profiles following tetraploidization

In order to investigate the effect of tetraploidization on primary metabolism, leaf samples of double diploid and diploid lines were analyzed by using an established GC-MS platform [37]. A total of 30 metabolites were identified by using an available chromatogram library. Utilizing the quantification internal standard, the content of every metabolite was calculated (Table 1).

**Table 1:** **24 of 30 primary metabolites were significantly accumulated in 4× Ziyang xiangcheng**

**Compound**	**2× (mean ± SE)** ^**a**^	**4× (mean ± SE)**	**Flod change**	**P-value**	**Trend** ^**b**^
**Sugars**					
Turanose	7.64 ± 0.37	9.65 ± 0.87	1.3		
Galactose	1.15 ± 0.16	5.97 ± 1.09	5.2	0.01	up
Fructose	41.48 ± 6.07	100.74 ± 4.67	2.4	0.01	up
Glucose	15.16 ± 1.54	55.49 ± 7.59	3.7	0.05	up
Sucrose	4403.79 ± 25.33	9472.04 ± 785.87	2.2	0.01	up
Glucopyranose	93.03 ± 6.78	86.91 ± 9.97	0.9		
Arabinose	36.55 ± 2.03	176.59 ± 29.03	4.8	0.01	up
Mannose	50.12 ± 2.86	185.1 ± 25.59	3.7	0.01	up
Myo-inositol	460.53 ± 12.61	634.93 ± 49.72	1.4	0.01	up
**Organic acids**					
Ethanedioic acid	21.9 ± 1.29	223.95 ± 16.25	10.2	0.01	up
Succinic acid	21.07 ± 2.24	26.3 ± 2.24	1.2		
citric acid	23.06 ± 1.75	98.79 ± 1.42	4.3	0.01	up
Isocitric acid	1132.63 ± 22.07	2067.81 ± 33.25	1.8	0.01	up
GABA	1.13 ± 0.04	35.55 ± 4.88	31.5	0.01	up
2-Ketoglutaric acid	63.8 ± 6.7	50.41 ± 0.79	0.8		
Malic acid	349.14 ± 42.51	1891.19 ± 90.58	5.4	0.01	up
2,3,4-Trihydroxybutyric acid	50.89 ± 2.74	235.16 ± 10.28	4.6	0.01	up
2-Keto-d-gluconic acid	8.45 ± 0.69	32.15 ± 1.17	3.8	0.01	up
**Amino acids**					
Glycine	4.23 ± 0.22	16.29 ± 1.45	3.9	0.01	up
Alanine	ND	11.59 ± 3.02			up
Threonine	ND	4.01 ± 0.61			up
Proline	ND	109.17 ± 14.01			up
Serine	ND	8.07 ± 1.75			up
Acetyl-lysine	ND	39.55 ± 3.48			up
**Fatty acids**					
Octadecanoic acid	77.69 ± 7.32	123.2 ± 6.8	1.6	0.01	up
Octadecanoic acid,2,3-bisoxypropylester	167.7 ± 13.83	352 ± 19.42	2.1	0.01	up
Hexadecanoic acid	19.53 ± 1.46	36.06 ± 2.05	1.8	0.01	up
Hexadecanoic acid,2,3-bisoxypropylester	41.66 ± 3.67	55.75 ± 5.24	1.3		
**Alcohols**					
Glycerol	204.19 ± 13.38	559.62 ± 63.31	3.3	0.01	up
Rhamnitol	61.76 ± 5.93	73.17 ± 3.98	1.2		

The quantities of metabolites were analyzed using GC-MS, and their levels were normalized to ribitol and calculated as ug per g fresh weight of leaves. The data presented represent mean ± SE of six biological repetitions of leaves collected from eight plants per line. ^a^ND represents the metabolite was not detected due to low concentration. ^b^Up represents the metabolite is up-regulated in 4× as compared to 2× (Student’s *t*-test).

**Figure 2:**
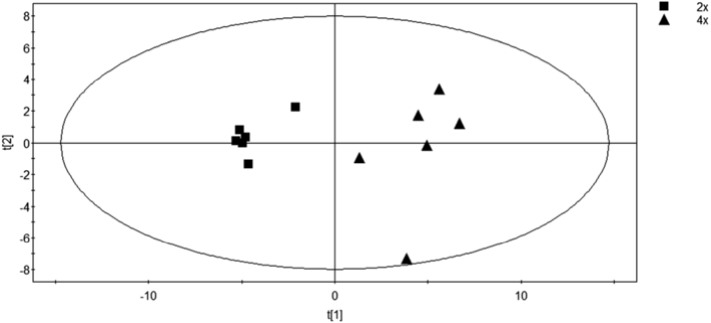
**Principal component analysis of GC-MS metabolite profiling data from 4× and 2× leaves.** First two components could explain 75.8% of the metabolite variance. Component 1 explained 58.6% of the variance and component 2 explained 17.2%.

Principal component analysis (PCA) served as an unsupervised statistical method to study the differences of the major metabolites of 4× and 2× (Figure 2). Parameters of the PCA model based on the primary metabolic data were: two principle components were calculated by cross validation, 58.6% of variables can be explained by first component and 17.2% of variables can be explained by the second component. A clear separation trend could be observed in the score plot (Figure 2), implying that extensive changes in the major metabolites were induced by tetraploidization.

Among the 30 metabolites, the levels of 24 metabolites in 4× leaves were significantly higher than those in 2×. But no significant changes in the rest 4 metabolites were observed. This indicated that tetraploidization has an activation effect on the accumulation of primary metabolites in leaves. Seven sugars were significantly accumulated in 4× (Table 1). It should be noted that in 4×, there was a 2.2-fold increase in the content of sucrose, which was the main sugar. Seven of nine identified organic acids exhibited 1.8- and 10.2-fold higher concentrations (Table 1), including γ-aminobutyric acid (GABA). Six amino acids, namely, glycine, alanine, threonine, proline, serine, and lysine, were detected in 4×, while only one amino acid, namely, glycine was detected in 2×. In addition, the content of three fatty acids and one alcohol in 4× increased (Table 1).

### Changes of secondary metabolic profiles following tetraploidization

**Figure 3:**
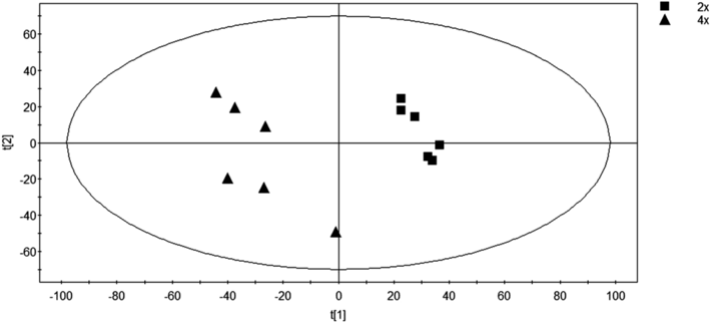
**Principal component analysis of LC-QTOF-MS metabolite profiling data from 4× and 2× leaves.** First two components could explain 49.3% of metabolite variance. Component 1 explained 32.8% of the variance and component 2 explained 16.5%.

To test whether the alteration of the ploidy has an influence on the level of leaf secondary metabolism, we performed non-targeted metabolite analysis using LC-QTOF-MS metabolomics technologies. In total, 3254 mass signals were detected in positive mode. PCA was performed to promote the classification of the metabolic phenotypes and the identification of the differential metabolites. The PCA effectively clusters biological replicates of the metabolomes of 2× and 4× into two categories, demonstrating extensive changes in the secondary metabolism caused by tetraploidization (Figure 3). Of these mass signals, 898 mass signals were significantly different between 4× and 2× (corrected p-value <0.05). 196 signals were up-regulated, and 702 signals were down-regulated in 4×, reflecting a decreased trend of secondary metabolite accumulation in 4×.

Significantly changed metabolites were analyzed by LC-ESI/MS/MS to obtain structure information. A total of 9 metabolites, namely, narirutin, naringin, hesperidin, neohesperidin, didymin, sinensetin, limonin, nobiletin and nomilin were identified by matching their mass spectra and retention time with known standards. The other 34 metabolites were tentatively identified according to ESI-MS fragmentation patterns (Table 2). These identified metabolites were mainly comprised of phenolic flavonoids, including 6 flavanones and 33 flavones. These flavones were mainly made up of polymethoxyflavones (PMFs), which are widely distributed in citrus. These identified metabolites also included an aromatic amine (octopamine), a cinnamic acid (coumaric acid) and two limonoids (limonin and nomilin). Notably, all the 33 identified flavones were down-regulated in 4×, while all the 6 flavanones were up-regulated.

**Table 2:** **Identified metabolites showing statistically significant changes between 2× and 4× Ziyang xiangcheng**

**Peak no.**	**RT (min)**	**Component name**	**[M + H]** ^**+**^	**MS/MS fragments**	**Family**	**Trend** ^**c**^
1	1.0	Octopamine^b^	154	91	Alkaloid	up
2	5.2	Coumaric acid^b^	165	147/120/65/91	Cinnamic Acid	down
3	8.8	Narirutin^a^	581	273/434	Flavanone	up
4	8.9	Neodiosmin^b^	609	301/463	Flavone	down
5	9.0	Naringin^a^	581	273	Flavanone	up
6	9.1	Hesperidin^a^	611	303	Flavanone	up
7	9.3	Neohesperidin^a^	611	303	Flavanone	up
8	9.9	Brutieridin^b^	755	303	Flavanone	up
9	10.5	Didymin^a^	595	287	Flavanone	up
10	10.6	PMFs-1^b^	359	344/329/298	Flavone	down
11	11.2	PMFs-2^b^	359	329/344	Flavone	down
12	11.5	PMFs-3^b^	375	360/345	Flavone	down
13	11.6	PMFs-4^b^	359	298/326/344	Flavone	down
14	11.8	PMFs-5^b^	359	326/344	Flavone	down
15	12.4	PMFs-6^b^	389	359/341/374	Flavone	down
16	12.5	PMFs-7^b^	359	329/344	Flavone	down
17	12.5	Isosinensetin^b^	373	343	Flavone	down
18	12.7	PMFs-8^b^	403	373/388	Flavone	down
19	12.8	PMFs-9^b^	389	359/374	Flavone	down
20	12.8	PMFs-10^b^	375	317/342	Flavone	down
21	12.9	PMFs-11^b^	419	389/371/404	Flavone	down
22	13.0	PMFs-12^b^	403	373/327/388	Flavone	down
23	13.1	PMFs-13^b^	345	330/315	Flavone	down
24	13.3	Sinensetin^a^	373	343/312/329/357	Flavone	down
25	13.3	PMFs-14^b^	343	328/313	Flavone	down
26	13.6	PMFs-15^b^	345	330/284/312	Flavone	down
27	13.6	PMFs-16^b^	405	375/390	Flavone	down
28	13.7	Limonin^a^	471	161/425	Limonoid	up
29	13.7	PMFs-17^b^	375	360/345/317	Flavone	down
30	14.0	PMFs-18^b^	359	343/329	Flavone	down
31	14.2	Nomilin^a^	515	161	Limonoid	down
32	14.2	Nobiletin^a^	403	373	Flavone	down
33	14.3	Tetramethyl-O-scutellarein^b^	343	313/282/299	Flavone	down
34	14.4	PMFs-19^b^	389	331/356/313/374	Flavone	down
35	14.5	PMFs-20^b^	359	329/346	Flavone	down
36	14.8	Heptamethoxyflavone^b^	433	418/403	Flavone	down
37	15.0	PMFs-21^b^	343	313/328	Flavone	down
38	15.0	PMFs-22^b^	359	298/326/343	Flavone	down
39	15.2	PMFs-23^b^	419	389/404	Flavone	down
40	15.8	PMFs-24^b^	405	375/347/357/390	Flavone	down
41	15.9	PMFs-25^b^	389	359/374/341	Flavone	down
42	16.5	PMFs-26^b^	419	389/404	Flavone	down
43	16.9	5-Demethyl tangeretin^b^	359	344/329/301	Flavone	down

[M + H]^+^, protonated molecular ion. ^a^Identified by matching their retention time and mass spectra with known standard. ^b^Putatively identified using ESI-MS fragmentation patterns. ^c^Relative increased (up) or decreased (down) concentration in 4× as compared to 2×. Student’s *t*-test was used and a p-value of less than 0.05 was considered significant. PMFs, polymethoxyflavones.

### Global transcriptome analysis

To investigate global transcriptome changes caused by tetraploidization, four cDNA libraries of 2× and 4× mature leaves were constructed. These libraries were sequenced by Illumina Hiseq 2500 platform. And 50 bp single-end reads were then generated. In total, 25,860,712 raw reads were generated from 2× and a total of 24,428,874 raw reads came from 4× (Additional file 5). After we removed reads containing adapter, reads containing poly-N, and low quality reads from raw data, 25,830,902 and 24,402,540 clean reads remained in 2× and 4×, respectively. The GC-contents were 43.30% in 2× and 43.16% in 4× respectively. To assess the sequencing quality, the reads were mapped to the *Citrus sinensis* reference genome. Of the two groups of duplicate data, 11,115,785 (86.06%) and 11,383,064 (88.14%) reads successfully mapped were generated from 2×-1-2×-2 and 11,250,774 (88.87%) and 10,531,271(89.69%) reads from 4×-1-4×-2 (Additional file 6).

More than 50% of the genes were expressed at a low level (<3 RPKM) and less than 8% of genes were expressed at a high level (>15 RPKM) in all samples (Additional file 7). Notably, there were no obvious differences between 2× and 4× in the percentage of genes at low, medium and high expression levels. This suggested tetraploidization didn’t have an effect on the inhibition or activation of gene expression.

Genes with an adjusted p-value <0.05 found by DESeq (R package, version 1.10.1) were assigned as differentially expressed. Totally 24073 genes were detected in all samples, while only 212 genes (0.8% of detected genes) were significantly differentially expressed between 2× and 4× seedling leaves. Of 212 DEGs, 96 genes were up-regulated and 116 genes were down-regulated in 4×, relative to 2×. For up-regulated genes, differences ranged from1.4-fold to 12.5- fold; for down-regulated genes, differences ranged from 1.4-fold and 13.4- fold. These results indicated that the range of gene expression changes between 2× and 4× was very limited.

**Figure 4:**
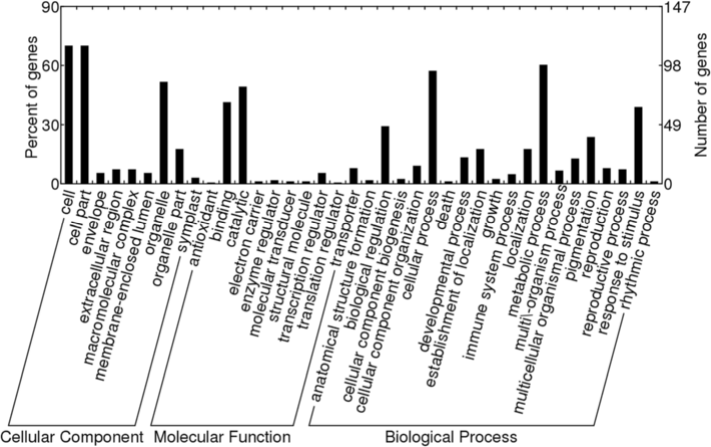
**GO categories of the DEGs between 2× and 4× Ziyang xiangcheng.** 163 out of the 212 DEGs were assigned to 957 GO annotations, which were divided into three categories: biological processes, cellular components, and molecular functions.

The functional gene ontology annotation of these DEGs was further performed by using Blast2Go software. 163 out of the 212 DEGs were assigned to at least one term in GO biological process, cellular component, and molecular function categories. Then the DEGs were classified into 38 subcategories in terms of function, almost covering all important categories of biological processes and molecular functions (Figure 4). In the biological process category, metabolic process and cellular process were the two largest groups, suggesting that extensive metabolic activities were taking place in 4× leaves. In the cellular component category, cell and cell part represented two major sub-categories, while catalytic and binding were dominant in molecular function category.

**Figure 5:**
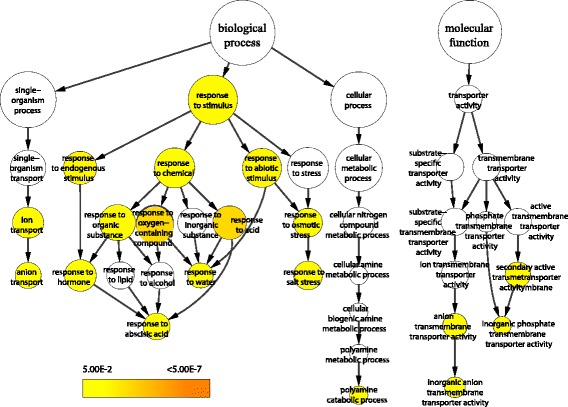
**Significantly enriched GO categories in DEGs between 2× and 4× Ziyang xiangcheng.** The colored nodes represent the significantly over-represented GO terms. The colored bar shows the significance.

GO enrichment analysis was performed by using BiNGO [38]. In biological process category, DEGs were found to be highly related to stress-response functions, such as response to salt stress, to water, and to abscisic acid (Figure 5). This indicated that some processes related to stress were induced in response to tetraploidization. The other two functions, namely anion transport and polyamine catabolic process, were also significantly enriched (Figure 5). In molecular function category, only two terms were overrepresented, namely, inorganic anion transmembrane transporter activity, inorganic phosphate transmembrane transporter activity (Figure 5). In cellular component category, no terms were overrepresented.

To identify the biological pathways in which the DEGs were involved, we mapped DEGs to the reference canonical pathways in KEGG. In total, 40 out of 212 DEGs were assigned to 46 KEGG pathways. The two largest clusters were metabolic pathways with 19 members and biosynthesis of secondary metabolites with 13 members (Additional file 8). It indicated that many DEGs involved in metabolic process in 4×. However, no KEGG terms was over-represented in DEGs.

**Figure 6:**
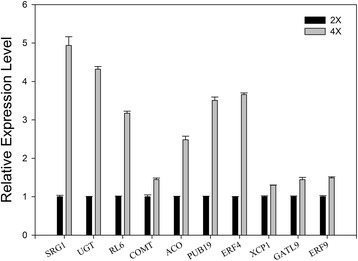
**Expression analysis of top 10 up-regulated functionally characterized DEGs in 4× Ziyang xiangcheng by qPCR.**

To validate the RNA-seq data, the following top 10 up-regulated functionally characterized genes were selected for qPCR assays: Fe(II)/ascorbate oxidase (SRG1, Cs9g09290), UDP-glucoronosyl/UDP-glucosyltransferase family protein (UGT, Cs5g11620), myb family transcription factor (RL6, Cs3g24870), caffeic acid O-methyltransferase (COMT, orange1.1 t02085), aminocyclopropane 1-carboxylic acid oxidase (ACO, Cs9g08990), u-box armadillo repeat protein (PUB19, Cs7g08470), ethylene response factor (ERF4, Cs1g07950), tracheary element vacuolar protein (XCP1, Cs2g27860), glycosyltransferase (GATL9, Cs7g07900), ethylene response factor (ERF9, Cs2g05620) (Additional file 9). As shown in Figure 6, all the 10 genes were verified to be up-regulated by qPCR analysis, although their fold changes differed from the result of RNA-seq. Notably, six of these genes, namely, SRG1 [39], COMT [40], ACO [41], PUB19 [42], ERF4 [43] and ERF9 [44] were involved in abiotic stress response.

## Discussion

### Stress related metabolites were significantly up-regulated in doubled diploid Ziyang xiangcheng

Metabolic alterations induced by tetraploidization might confer plant a better adaptation to environmental stresses. Primary metabolites are required for growth, development and interactions of plants with their environment [45]. In this study, most of the detected primary metabolites were up-regulated in 4× Ziyang xiangcheng (Table 1). It indicated that tetraploidization had an activation effect on primary metabolism. These up-regulated metabolites include sugars, amino acids, organic acids, and fatty acids. Notably, these metabolites play an important role during plant adaptations to environmental stresses.

Sugars are involved in various abiotic stresses. They have several functions in plants suffering abiotic stresses: acting as osmoprotectants to maintain osmotic balance and stabilize macromolecules or as metabolite signaling molecules to activate specific signal transduction pathway, and providing energy source to recover from water deficit [46,47]. Accumulation of sugars is strongly correlated with improved plant stress tolerance to drought stress [46,48,49]. For example, sucrose accumulates in almost all desiccation-tolerant flowering plants [50] and fern [51]. In this study, seven out of nine detected sugars including sucrose, glucose and fructose were up-regulated in 4×, which implied 4× might have advantages over 2× under drought stress.

A case in point is that increased levels of proline correlate with enhanced stress tolerance [48,52]. Proline was considered to have several functions under stress conditions, including osmotic adjusting, reactive oxygen species (ROS) scavenger and protection of proteins from denaturation [52-54]. Therefore, higher concentration of proline might promote abiotic stress tolerance in 4×. Additionally, Yobi et al. [55] found that desiccation-tolerant species *Selaginella lepidophylla* had significantly higher concentration of sugars, sugar alcohols and amino acids than desiccation-sensitive species *Selaginella moellendorffii*. Compared to 2×, higher concentration of stress metabolites in 4× might be also beneficial for the cultivar grafted on it. A study performed on Rangpur lime (*Citrus limonia*) rootstock demonstrated that tetraploids increase drought tolerance via enhanced constitutive root abscisic acid production [26]. In that study, diploid and tetraploid clones of Rangpur lime rootstocks were grafted with 2× Valencia Delta sweet orange (*Citrus sinensis*) scions, named V/2×RL and V/4×RL, respectively; V/4×RL leaves had greater abscisic acid (ABA) content under normal condition, compared to V/2×RL [26]. Studies of *Arabidopsis* polyploids revealed that the content of leaf potassium and rubidium was evaluated in in diploid leaves on shoots grafted to tetraploid roots, whereas leaves from tetraploid shoots grafted to diploid roots showed the same leaf K as diploid [13]. So we may presume that a distinct metabolic phenotype would be observed between the scion cultivars grafted on 4× and 2× Ziyang xiangcheng respectively. Higher content of stress-related metabolites in 4× might be beneficial for the cultivar grafted on it. In addition, tetraploid rootstock may also have a dwarfing effect on scion cultivar being grafted on it, compared with the diploid rootstock [56].

### Gene expression divergence caused by tetraploidization is involved with stress response

A small genome expression change was observed between diploid and autotetraploid according to studies performed on several species. In *Paspalum notatum* and *Isatis indigotica*, about 0.6% and 4% variations in transcript abundance were detected between diploid and autotetraploid by using the *Arabidopsis thaliana* whole genome gene chip [57,58]. In *Arabidopsis thaliana* Col-0 ecotype and Ler-0 ecotype, Yu et al. [25] found about 1% and 0.1% variations between diploid and autotetraploid, respectively. We found less than 1% genes were differentially expressed between diploid and doubled diploid Ziyang xiangcheng. A similar number of genes were also detected between diploid and tetraploid *Citrus limonia* [26]. These studies altogether with our study suggested that the effect of genome doubling on gene expression is relatively limited. Here, we should point out that the 4× Ziyang xiangcheng came from doubling a hybrid (*C. ichangensis* × *C. reticulata*). Theoretically, the doubled diploid should be an allotetraploid rather than an autotetraploid (doubling a homozygous diploid) [6]. Therefore, the expression pattern of doubled diploid Ziyang xiangcheng should consist with the one of an allotetraploid rather than the one of an autotetraploid**.** Genome expression changes in allotetraploids are considered to be more strongly affected by genome hybridization than by changes in ploidy levels [19,59]. So we presume that a relatively large change in genome expression could be detected between doubled diploid Ziyang xiangcheng and its putative parents (*C. ichangensis* and *C. reticulata*). Herein, we only focused on the effect of genome doubling on gene expression.

Genes involved in the response to abscisic acid and abiotic stimulus, were differentially expressed following genome doubling according to GO enrichment analysis (Figure 5). This indicates that 4× Ziyang xiangcheng might be able to respond to abiotic stresses in a flexible and fast way, to some extent [14]. Interestingly, the phenomenon that tetraploidization influences the expression of genes involved in hormone and abiotic stress responses was also reported in autotetraploid *A. thaliana* [14,25]. We also found that the expression of genes involved in ion transport was also affected by genome doubling. It is known that ion transport is highly related to salt tolerance [60].

Higher potassium accumulation and salinity tolerance has been found in *Arabidopsis* polyploids [13]. The higher potassium accumulation might be partly due to altered expression of genes involved in ion transport.

Moreover, six out of ten top up-regulated genes were involved in ABA- and stress-related process (Additional file 9). The first gene, namely SRG1, was associated with senescence-related processes, encoding a member of the Fe(ll)/ascorbate oxidase superfamily protein, and its expression was induced under drought and heat stress [61,62]. Caffeic acid O-methyltransferases encoded by COMT genes are key enzymes of lignin biosynthesis [63], affecting cell wall structure, and COMT was up-regulated by drought stress in maize [40]. ACO genes encode 1-aminocyclopropane-1-carboxylate (ACC) oxidases which catalyze the reaction from ACC to ethylene [64], and water stress induced ACO gene expression in sunflower leaves was previously reported [65]. PUB19 encodes a U-Box E3 ubiquitin ligase and it was up-regulated by drought, salt, and cold stress and ABA [42]. The last two genes, namely ERF4 and ERF9, which are the members of the ERF/AP2 transcription factor family, are involved in various reactions to abiotic stresses [66]; these two genes bind to the GCC box, DRE/CRT, CE1 elements, and they acted as repressors of gene transcription, enhancing plant tolerance to multiple stresses [67]. Overexpression of ERF4 gene increased tolerance to salt and drought stress in *Arabidopsis* [66]. These reports, together with our results suggest 4× Ziyang xiangcheng may be pre-adapted to abiotic stresses, compared to 2×.

### The transcriptome divergence cannot explain the metabolic changes

In order to integrate leaf transcriptome data with the metabolic profiling, attention was focused on the DEGs involved in metabolic pathway. Among these DEGs, 40 were assigned to 46 pathways and no significantly enriched KEEG pathways were found. It implies that the limited DEGs involve in a wide range of pathways, but their functions are dispersive.

To a great extent, the accumulation pattern of the DEGs encoding proteins or enzymes involved in metabolic processes was not consistent with the differences observed in the metabolite profiling (Additional file 10). Most of the detected sugars, amino acids and fatty acids were significantly accumulated in 4×. However, most of the genes involved in these metabolic processes were down-regulated in 4×. For example, the sucrose content of 4x leaves was 2-fold than that of 2×. But the gene encoding sucrose synthase was significantly down-regulated in 4×. In another example, in flavone and flavonol biosynthesis, only one gene, namely, COMT was differentially expressed between 4× and 2×. The gene encoding a caffeic acid O-methyltransferase, positively regulates flavonoid biosynthetic process and may be involved in PMFs (polymethoxyflavones) synthesis [68]. Theoretically, the up-regulation of COMT should promote the accumulation of PMFs in 4×. However, all detected PMFs were down-regulated in 4×. The discordance between transcriptomic and metabolomic data is probably related to several factors. First, it is not easy to find a strict correlation between metabolite accumulation and gene expression because of the complexity in metabolic networks [69,70]. Second, small RNAs, including microRNAs and small interfering RNAs might play an important role in some gene regulation [71]. Third, reactivation of transposable elements (TEs) following polyploidization in synthetic hexaploid wheats (*Triticum*) was considered to participate in regulation of the transcription of neighbouring genes [72]. At last, post-translational modifications may contribute to the discordance between transcriptomic and metabolomic data. The transcriptome divergence might not reflect the protein divergence between 4× and 2× Ziyang xiangcheng, leading to the discordance. In support of this hypothesis, percentage of differentially accumulated proteins between autotetraploid and diploid *Arabidopsis thaliana* that matched the differentially expressed genes was relatively low, due to post-transcriptional regulation and translational modifications of proteins during polyploidization [73]. Similarly, transcriptional changes do not explain differential protein regulation in resynthesized *Brassica napus* allotetraploids [74].

## Conclusions

Our results suggest that tetraploidization has multi-level effects on Ziyang xiangcheng. Morphological and anatomical traits like leaf thickness, stoma number, stomatal density and vessel size were altered as a consequence of tetraploidization. The metabolic phenotype was also significantly altered following tetraploidization and many stress-related metabolites, such as sucrose, proline and GABA were significantly up-regulated in 4×. However, relatively small transcriptome alterations were induced by tetraploidization. Notably, the transcriptome alterations were highly related to hormone and stress responses, and many top up-regulated genes in 4× were associated with stress response. Interestingly, the transcriptional divergence could not adequately explain the metabolic changes, probably due to post-transcriptional regulation. Compared to diploid, higher expression level of stress related genes and higher content of stress related metabolites in doubled diploid could be beneficial for its stress tolerance. Our data will help better understanding the consequences caused by polyploidization and be valuable for citrus rootstock breeding in the future.

## Methods

### Plant materials

Seeds of diploid (2n = 2× = 18) Ziyang xiangcheng (*Citrus junos* Sieb. ex Tanaka) were collected and provided by Professor Keling Chen, Institute of Horticulture Research, Sichuan Provincial Academy of Agricultural Science, China. Seeds were grown in pots filled with commercial soil in the greenhouse. Doubled diploid and diploid Ziyang xiangcheng were prepared by Liang et al. [30]. Thirteen uniform 1-year-old 2× and eight uniform 1-year-old 4× seedlings were respectively selected and transplanted in commercial soil. Seedlings were grown in the greenhouse under natural photoperiod conditions and they were irrigated twice a week. The ploidy levels of these seedlings were determined by flow cytometry (CyFlow Space, Partec, Germany). The genetic constitution of these seedlings was further analyzed by the published SSR markers [75,76], namely, Ci01C09, Ci06A05b, Ci07C07, Ci07E06, mCrCIR08A03, CCSM40, CCSM46, CCSM69.

### Sampling

Fully expended leaves (from fourth or fifth leaf from the top, 3–5 month old, from the spring flush of the current season) were collected in the morning. The leaves being used for metabolic and transcriptional profiling were immediately frozen in liquid nitrogen and stored at −80°C.

### Morphological and anatomic analysis

Leaf thickness and area was determined using a micrometer and a portable area meter (Yaxin-1241, Beijing), respectively. Three leaves of each individual seedling were measured. SEM analysis was performed using one leaf of each individual plant according to the method described by Yi et al. [77]. Photographs were taken to measure stomatal size and density. About 60 stomata were measured for each genotype.

Samples were fixed, dehydrated and embedded according to Hu et al. [78]. Transverse sections about 1–5 μm thick were cut using a Leica Ultracut R ultramicrotome (Leica, Bensheim, Germany). The sections were stained with Toluidine Blue O (Aldrich, Milwaukee) and photographed with a BX61 fluorescence microscope (Olympus, Tokyo). The morphometrical analysis was performed using Image-Pro Plus 6.0 software (Media Cybernetics, USA).

### The primary metabolic profiling

Six independent plants were used as biological replicates, and about five leaves were sampled from each plant in primary metabolic profiling. Non-targeted metabolite profiling was carried out by GC-MS using a modified method described by Yun et al. [37]. A total of 200 mg ground leaf samples were extracted in 2,700 μl methanol and ribitol solution (300 μl, 0.2 mg ml^−1^) was added as an internal standard. The samples were centrifuged, dried and derivatized. GC-MS analysis was performed by using a Thermo Trace GC Ultra, coupled with Thermo Fisher a DSQ II mass spectrometer (Thermo Fisher Scientific, Waltham, MA, USA). Metabolites were identified by using an available chromatogram library and PCA analysis was performed by using the software Simca-P (Ver 11, Umetrics, Umea, Sweden).

### The secondary metabolic profiling

Six independent plants were used as biological replicates, and about five leaves were sampled from each plant in secondary metabolic profiling. The secondary metabolic profiling was performed by LC-QTOF-MS using a modified method according to Yun et al. [37]. 100 mg freeze-dried powder was extracted with 80% methanol over night at 4°C. The mixture was centrifuged and filtered. Then, the metabolic profiling were performed using a QTOF 6520 mass spectrometer (Agilent Technologies, Palo Alto, CA, USA) coupled to a 1200 series Rapid Resolution HPLC system.

The raw data was processed by Agilent Mass Hunter Qualitative Analysis (version B. 04.00, Aglient Technologies) and Mass Profiler Software (version B.02.02, Aglient Technologies). Then PCA analysis was performed using the filtered and normalized data. Metabolite identification was carried out by comparing mass spectra and retention time with those of authentic standards. Nine authentic standards, namely, narirutin, naringin, hesperidin, neohesperidin, didymin, sinensetin, limonin, nomilin, and nobiletin were obtained from Sigma Chemical Co. (St. Louis, MO).

### RNA sequencing and data analysis

Leaves from four plants were pooled as an independent biological replicate and leaves from other four trees were pooled as the other independent biological replicate in transcriptomic analysis. Total RNA extraction and a quality assessment were performed according to the protocol described by Zheng et al. [79]. RNA Samples were sent to Novogene Bioinformatics Technology Co. Ltd (Beijing), where the libraries were constructed. Sequencing libraries were generated from 3 μg total RNA using NEBNext Ultra RNA Library Prep Kit (NEB, USA) and sequenced on an IlluminaHiseq 2500 platform and 50 bp single-end reads were generated.

Clean reads were obtained by removing reads containing adapter, reads containing ploy-N and low quality reads from raw data and were aligned to the *Citrus sinensis* genome [80] (http://211.69.128.148/orange/index.php) using TopHat (2.0.9) software [81]. To estimate gene expression level, RPKM of each gene was calculated based on the length of the gene and reads count mapped to this gene. Genes RPKM values were calculated based on all the uniquely mapped reads. The genes with RPKMs ranging from 0 to 3 were considered at a low expression level; the genes with RPKMs ranging from 3 to 15 at a medium expression level; and the genes with RPKMs above15 at high expression level.

Differential expression analysis was implemented using the DESeq R package (1.10.1) [82]. Genes with an adjusted P-value <0.05 found by DESeq were assigned as differentially expressed. GO (Gene Ontology) annotation was performed by using Blast2GO software (GO association done by a BLASTX against the NCBI NR database). Then GO enrichment analysis of differentially expressed genes was performed by the BiNGO plugin for Cytoscape [38]. Over-presented GO terms were identified by using a hypergeometric test with a significance threshold of 0.05 after a Benjamini and Hochberg FDR correction [83]. KEGG enrichment analysis of differentially expressed genes was performed by KOBAS (2.0) software [84].

### Verification of RNA-seq by q-PCR

To test the reliability of RNA-seq, a set of top ten up-regulated genes in 4× were selected for qRT-PCR. Specific primers were designed with the Primer Express software (Applied Biosystems) and synthesized by Sangon (Shanghai, China). The cDNA was synthesized from 1 μg of total RNA using PrimeScript RT reagent Kit (Takara, Dalian, China). Real-time RT–PCR was performed on the ABI 7500 Real-Time PCR System (Applied Biosystems) using the 2× SYBR green PCRmaster mix (Applied Biosystems). Three independent biological replicates were analyzed for each sample and data were indicated as mean ± SE (n = 3).

### Availability of supporting data

Raw sequencing data is available through the Gene Expression Omnibus (GEO) under accession NO. GSE65416 at website: http://www.ncbi.nlm.nih.gov/geo/query/acc.cgi?acc=GSE65416.

## Additional files

Additional file 1:
**SSR analysis of eight 4× and thirteen 2× Ziyang xiangcheng seedlings.** Black arrows represent seedlings possessed heterozygous loci. Additional file 2:
**Comparison between 2× and 4× Ziyang xiangcheng seedlings with respect to height, stem diameter, leaf area, stoma size and density.** ^a^An asterisk (*) indicates significant differences (Student’s *t*-test; P < 0.01). Additional file 3:
**Transversal sections of mature leaves (fourth or fifth leaf from the top) in 2× (A and C) and 4× (B and D) Ziyang xiangcheng.** Anatomy of leaf blades (A and B) and leaf central vein (C and D) were shown. Abe, abaxial epidermis; Ade, adaxial epidermis; Cut, cuticle; Is, intercellular space; Pa, parenchyma; Pi, pith; Ph, phloem; Pp, palisade parenchyma; Sc, sclerenchyma; Sp, spongy parenchyma; X, xylem. Additional file 4:
**Anatomical characteristics of leaves from 2× and 4× Ziyang xiangcheng seedlings.** ^a^An asterisk (*) indicates significant differences (Student’s *t*-test; P < 0.01). Additional file 5:
**Quality of illumina sequencing.**
Additional file 6:
**Summary of read mapping statistics.**
Additional file 7:
**Statistics of genes in different expression-level intervals.**
Additional file 8:
**KEGG classification of the DEGs between 2× and 4× Ziyang xiangcheng.** 44 out of the 212 DEGs were assigned to 46 KEGG pathways. The top 10 most abundant KEGG pathways are shown. Additional file 9:
**Primer sequences for Real-time PCR analysis.**
Additional file 10:
**DEGs involved in major metabolic pathways.**

## Abbreviations

2×: Diploid Ziyang xiangcheng
4×: Doubled diploid Ziyang xiangcheng
GAs: Glycoalkaloids
GC-MS: Gas chromatography coupled to mass spectrometry
LC- QTOF- MS: Liquid chromatography quadrupole time-of-flight mass spectrometry
PCA: Principal component analysis
GABA: γ-aminobutyric acid
PMFs: Polymethoxyflavones
DEGs: Differentially expressed genes
ROS: Reactive oxygen species
