# Supplementary figures and images for: Comparative metabolic and transcriptional analysis of a doubled diploid and its diploid citrus rootstock (C. junos cv. Ziyang xiangcheng) suggests its potential value for stress resistance improvement
Source: BMC Plant Biol. 2015 Mar 18;15:89. doi: 10.1186/s12870-015-0450-4 (PMC4374211; doi:10.1186/s12870-015-0450-4)

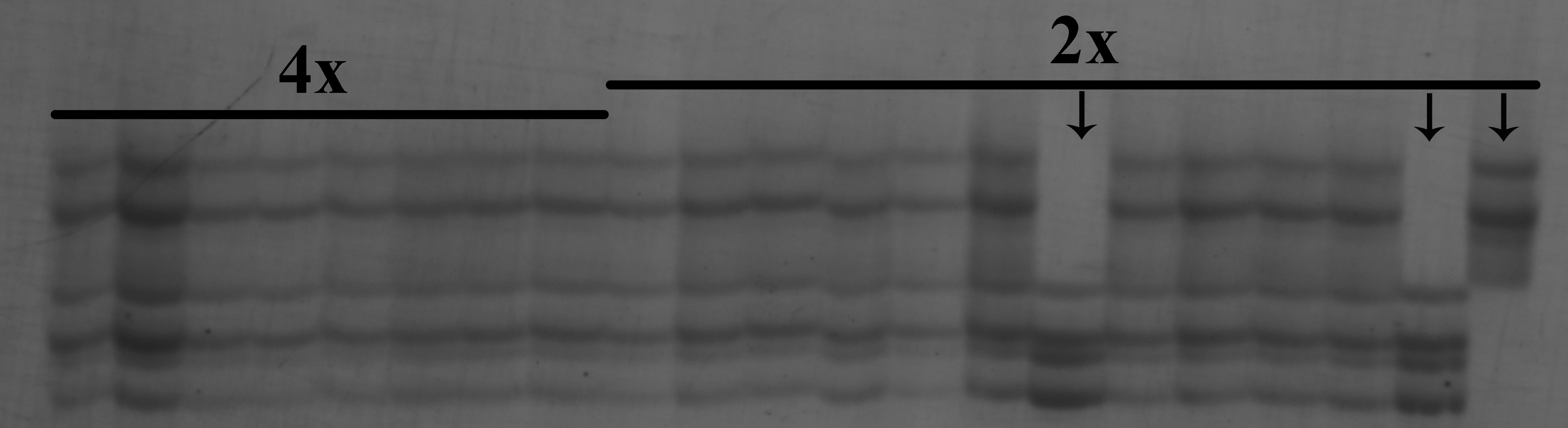

Supplement: Additional file 1: — SSR analysis of eight 4× and thirteen 2× Ziyang xiangcheng seedlings. Black arrows represent seedlings possessed heterozygous loci. [file 12870_2015_450_MOESM1_ESM.tiff]

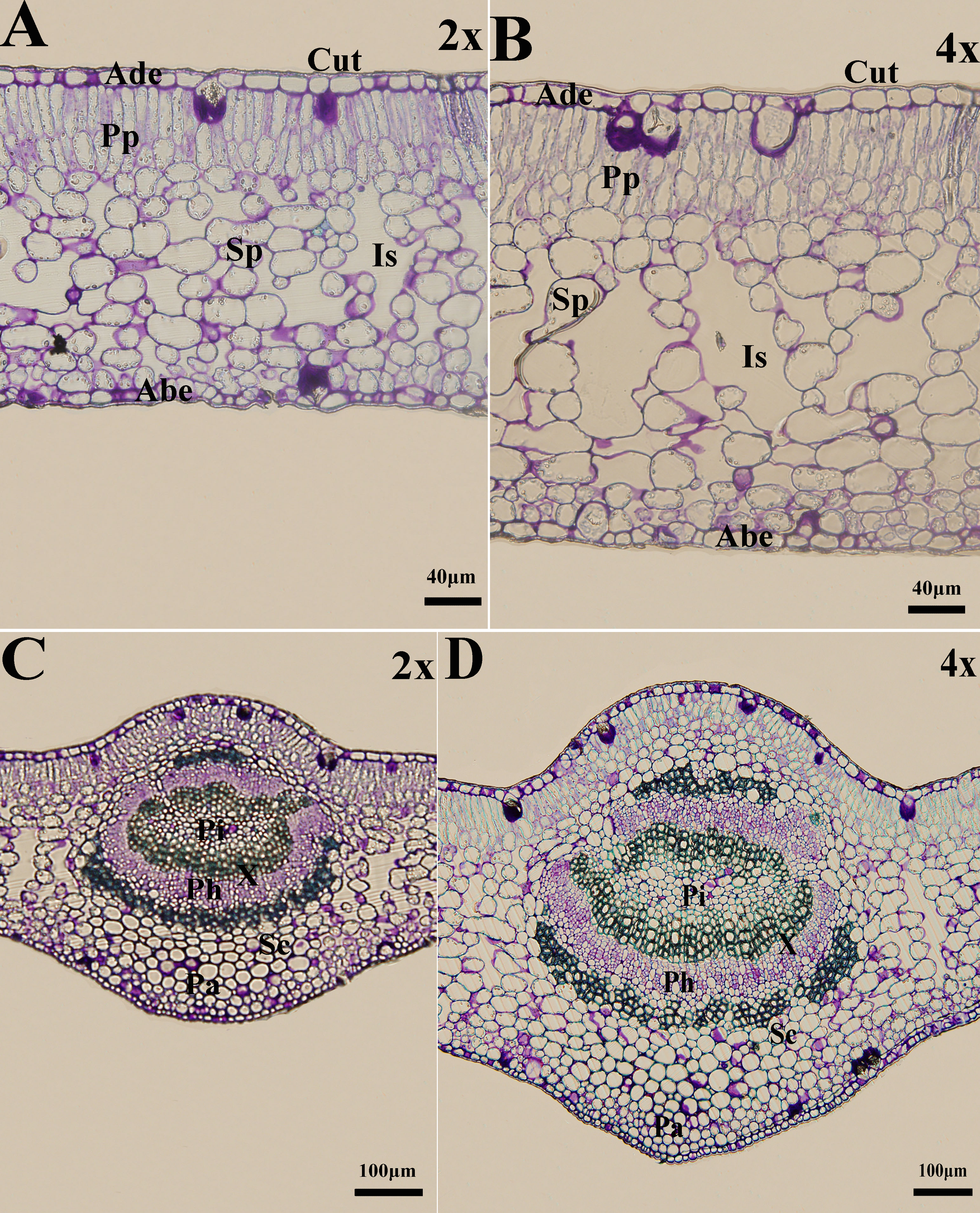

Supplement: Additional file 3: — Transversal sections of mature leaves (fourth or fifth leaf from the top) in 2× (A and C) and 4× (B and D) Ziyang xiangcheng. Anatomy of leaf blades (A and B) and leaf central vein (C and D) were shown. Abe, abaxial epidermis; Ade, adaxial epidermis; Cut, cuticle; Is, intercellular space; Pa, parenchyma; Pi, pith; Ph, phloem; Pp, palisade parenchyma; Sc, sclerenchyma; Sp, spongy parenchyma; X, xylem. [file 12870_2015_450_MOESM3_ESM.tiff]

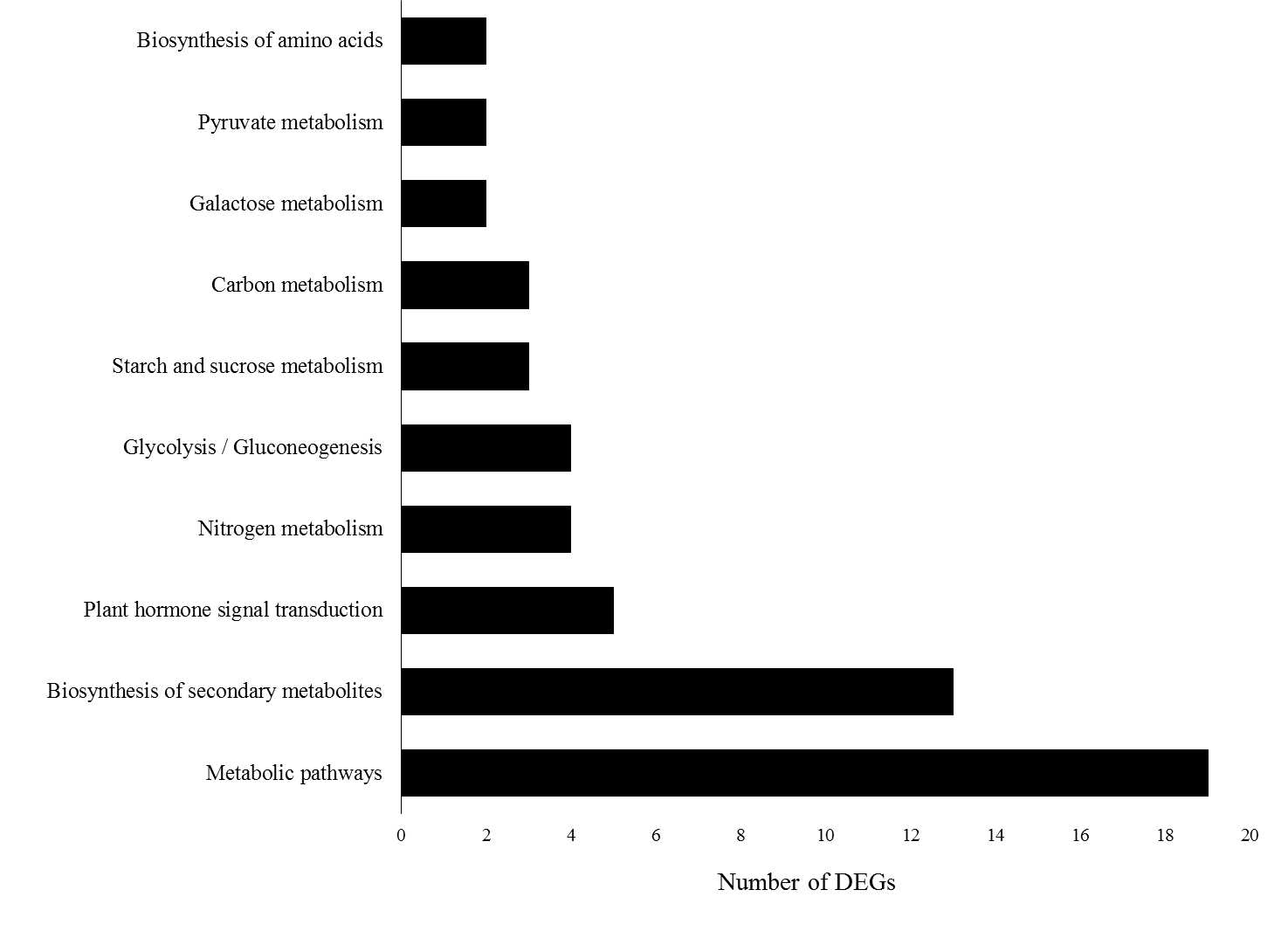

Supplement: Additional file 8: — KEGG classification of the DEGs between 2× and 4× Ziyang xiangcheng. 44 out of the 212 DEGs were assigned to 46 KEGG pathways. The top 10 most abundant KEGG pathways are shown. [file 12870_2015_450_MOESM8_ESM.png]
